# Innovation and renovation: *FEBS Open Bio* in 2023

**DOI:** 10.1002/2211-5463.13531

**Published:** 2023-01-03

**Authors:** Duncan E. Wright, Miguel A. De la Rosa

**Affiliations:** ^1^ FEBS Open Bio Editorial Office Cambridge UK; ^2^ Institute for Chemical Research (IIQ), Scientific Research Centre Isla de la Cartuja (cicCartuja) Universidad de Sevilla‐CSIC Spain

## Abstract

*FEBS Open Bio* is constantly evolving to best suit the needs of the scientific community. In this Editorial, we review the various new initiatives introduced in 2022 and look forward to the opportunities and challenges that lie ahead in 2023.

At the beginning of 2022, we faced the very difficult task of ensuring that *FEBS Open Bio* surpassed the high standards for innovation, quality and reliability set in 2021. With the invaluable and tireless support of our editorial board members, publishing partner, reviewers, authors, readers and colleagues, we are confident that we accomplished our goal. In 2022, we published four excellent “In the Limelight” issues, focussing on lysosomes, virology, the structures of SARS‐CoV‐2 proteins, and neurotransmitter release, respectively. We also introduced Research Protocols as a new article type, held the journal's first ever in‐person editorial board meeting, awarded 19 poster prizes at 15 international meetings in 10 different countries, and appointed five new editors and two new members of the Editorial Advisory Board. In this editorial, we summarise the new developments in this amazing year and look forward towards 2023.

## A warm welcome and fond farewell to new and departing editors

*FEBS Open Bio* receives submissions from all over the world on a broad range of subdisciplines, and we constantly strive to ensure that our editorial board reflects this diversity. In 2022, we were very pleased to appoint Francesco (Frank) Michelangeli (Emeritus Professor Biochemistry, University of Chester, UK; Honorary Professor in Biosciences, University of Birmingham, UK), Simon Rayner (Adjunct Professor, University of Oslo, Oslo, Norway), Hongxia Wang (Director, Comprehensive Cancer Center, Shanghai General Hospital, Shanghai Jiaotong University, Shanghai, China) and Ying Zhao (Professor, Department of Biochemistry and Molecular Biology, Peking University Health Science Center, Beijing, China) to the editorial board. In addition, we are delighted to welcome Manuel João Costa (Associate Professor, University of Minho, Braga, Portugal) to the journal's Education section. We would also like to congratulate Luciane V. Mello (Professor of Bioscience Education, University of Liverpool, UK) for her promotion to head of the Education section in 2022.
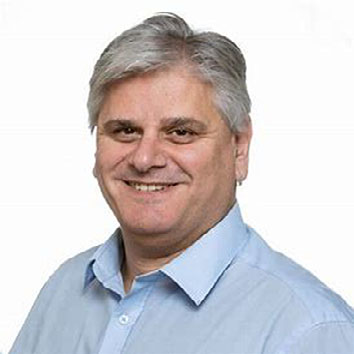


Francesco (Frank) Michelangeli is an Emeritus Professor of Biochemistry at the University of Chester and Honorary Professor in Biosciences at the University of Birmingham, UK. Frank gained his PhD from the University of Southampton and was subsequently awarded a NATO fellowship to undertake research into Ca^2+^ homeostasis mechanisms at the University of Padua, Italy. His main research interests are in membrane biochemistry and Ca^2+^ transport proteins. Frank was the honorary membership secretary and a trustee of the Biochemical Society, and is currently Treasurer of The Federation of European Biochemical Societies (FEBS).
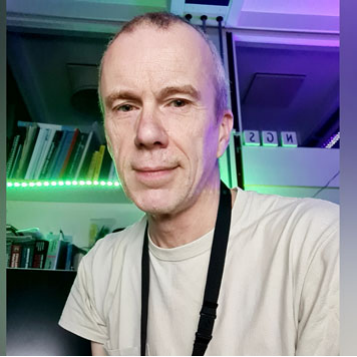


Simon Rayner is a Professor/Group Leader of the Non‐Coding RNA Research Group in the Department of Medical Genetics (DMG) at Oslo University Hospital (OUS)/University of Oslo (UiO). He is also a PI in the Organoid on Chip Centre of Excellence at UiO. His primary research interest is communicable and non‐communicable disease. For non‐communicable disease, he is studying the regulatory roles of the non‐coding genome and the association between ethnic variation and predisposition to Type 2 Diabetes. His work in infectious disease stems from his ties to China as a Chinese Academy of Sciences (CAS) senior scholar and through his previous position as a PI at Wuhan Institute of Virology, CAS.
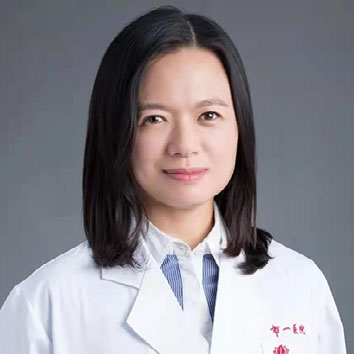


Hongxia Wang is the chief of the Department of Oncology and Chair of Comprehensive Cancer Center, Shanghai General Hospital, Shanghai Jiaotong University School of Medicine. Her research mainly focuses on the molecular mechanism of cancer stem cell and tumour microenvironment in tumour recurrence, metastasis, and treatment resistance. Another current focus of her group is to improve the clinical application of liquid biopsies in patients with breast cancer, including CTCs and tumour‐derived exosomes. In addition, her team has undertaken large, population‐based studies to examine the use of novel drugs and technologies for cancer patients, especially breast cancer and pancreatic adenocarcinoma.
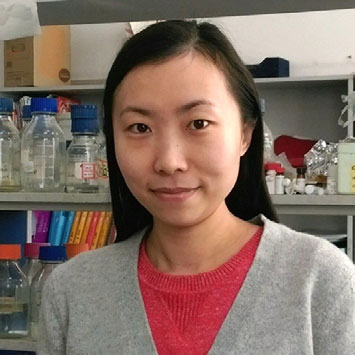


Ying Zhao completed her PhD at Peking University Health Science Center in Beijing, China, and she is now a professor in the Department of Biochemistry and Molecular Biology, School of Basic Medical Sciences, Peking University Health Science Center. Her research focuses on autophagy and the tumour microenvironment, and she has published several papers in *Nature Cancer*, *Nature Cell Biology*, *Molecular Cell*, *Cell Research*, and other journals.
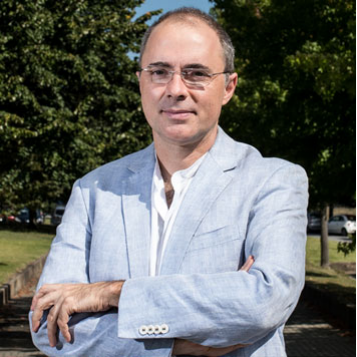


Manuel João Costa is an Associate Professor at the School of Medicine and Pro‐Rector for Educational Innovation and Student Affairs at the University of Minho. He is also a coordinator of the University of Minho's centre for innovation and development to teaching and learning (IDEA‐UMinho). He has been appointed to the Learning and Teaching Steering Committee of the European University Association and the Education Committee of the Federation of European Biochemical Societies (FEBS), and is also a delegate at the faculty development committee of the Association for Medical Education in Europe. In addition to *FEBS Open Bio*, Manuel also serves on the editorial board of *Biochemistry and Molecular Biology Education*, and previously served as an associate editor for *PLOS One* and *BMC Medical Education*.

We would also like to offer our heartfelt thanks and appreciation to three editors who stepped down from the board in 2022: Alexander Gabibov (Professor, Shemyakin–Ovchinnikov Institute of Bioinorganic Chemistry, Moscow, Russian Federation), Angel Herráez (Associate Professor, University of Alcalá, Spain) and Michelle Hill (Honorary Associate Professor, University of Queensland Centre for Clinical Research, Faculty of Medicine, University of Queensland). We are extremely grateful for their many years of service on the editorial board and wish them all the best for the future. Angel Herráez was one of the original editors appointed to helm the Education section on its founding in 2017, and he and Luciane V. Mello were instrumental in the successful growth and development of this section.

The journal's Editorial Advisory Board (EAB) support our editors in providing thorough, timely, fair and expert peer review. In 2022, we were pleased to welcome two new members to the EAB:

- Janesh Kumar
  National Centre for Cell Science (NCCS), Pune, India
- Tsukasa Tominari
  National Institute of Neuroscience, National Center of Neurology and Psychiatry, Tokyo, Japan

In 2022, we also said goodbye to Jacob Weller, who worked on *FEBS Open Bio* and *Molecular Oncology* as the journals' Editorial Assistant from 2018 to the beginning of 2022. We will miss Jacob's professionalism, hard work and good humour, and wish him well in his new role at Frontiers. Jacob is succeeded in the role by Irene Alvarez‐Domenech, who has quickly adapted to the role and has become a highly valued member of the team. We would also like to congratulate Ruzhica Bogeska on her well‐deserved promotion to Senior Editor.

## New developments in 2022

### In the Limelight issues and webinars

In 2021, *FEBS Open Bio* started to publish special issues containing a number of Review articles focussed on a particular topic. In 2022, we published four ‘In the Limelight’ issues, centred on lysosomes, virology, the structures of SARS‐CoV‐2 proteins and neurotransmitter release, respectively.

Jonathan Martinez‐Fabregas, a member of the journal's EAB, served as a guest editor of our special lysosomes issue. This issue contained five Review articles focussed on the important role of lysosomes, once thought to be little more than waste disposal units, in health and disease: in their article, Jonathan Martinez‐Fabregas, Irene Díaz‐Moreno and colleagues indicated that the lysosome is part of a complex communication network between organelles, and lysosome dysfunction is linked to a variety of pathologies [1]. These disease states include cancer and neurodegeneration, as reviewed by Janko Kos and co‐authors in the same issue [2]. Lysosomes are also implicated in ageing‐related diseases through cross‐talk between the autophagy‐lysosome pathway and mTORC1 activity; Timothy J. Sargeant and colleagues explored this interaction and whether targeting this pathway might slow the ageing process [3]. Thomas Reinheckel and Martina Tholen reviewed how the release of cathepsin proteases from lysosomes may not be a death sentence for the cell, and that cathepsins have other cellular functions compatible with cell survival [4]. Finally, Colin Watts reviewed the key role of lysosomes and lysosome‐related organelles in innate and adaptive immunity [5].

Our second special issue in 2022, published in June, took a broad look at virology under the direction of guest editor Marcelo López‐Lastra. Encarnacion Martinez‐Salas and co‐authors provided an overview of the strategies used by picornaviruses to hijack the cellular machinery to translate viral proteins [6]. In the second review, Pablo A. González and co‐authors explored the role of HSF1 in viral infections, and whether positive or negative modulation of this protein may have potential for the treatment of viral infections [7]. Recent years have reminded all of us of the importance of the continued development of effective antiviral agents, and C. Joaquin Caceres, Daniel R. Perez, and co‐authors provided a timely summary of the approved influenza antivirals, antiviral strategies under evaluation in clinical trials, and preclinical evaluations of novel compounds effective against influenza in animal models [8]. Understanding the host's own immune response to viral infection is also key to prevention and treatment, and Andrea Cimarelli and co‐authors concluded the issue by discussing the mechanisms by which ISG20 inhibits a broad spectrum of viruses [9].

Our third special issue of the year placed the limelight firmly on one specific virus: SARS‐CoV‐2. Guest editor Alexander Wlodawer commissioned three Review articles focussing on the structures of different proteins of SARS‐CoV‐2: Robin Stanley and co‐authors discussed the structure and function of ribonucleases [10]; Franck Martin and colleagues focussed on viral and cellular translation during SARS‐CoV‐2 infection, as mediated by NSP1 [11]; and finally, Xinquan Wang, Jiwan Ge and co‐authors discussed the evolution of and therapeutic targeting of the spike glycoprotein [12].

We are constantly striving to ensure that our published content is as accessible and prominent as possible. In November 2022, we ventured into a new medium to disseminate our content by hosting a free webinar to accompany our In the Limelight issue on SARS‐CoV‐2 protein structure. Alex Wlodawer, Robin Stanley, Franck Martin and Xinquan Wang participated in a roundtable discussion on important advances, ongoing challenges and unanswered questions in the field of coronavirus protein structure, including how changes in the structure of the spike protein affect virus activity. This fascinating webinar was very well attended, and we look forward to hosting webinars on other important topics in future for the benefit of the scientific community. We encourage those who were unable to attend to listen to the recording on the journal website here: https://febs.onlinelibrary.wiley.com/journal/22115463/webinar.

Our final In the Limelight issue of 2022 focussed on neurotransmitter release: guest editor Josep Rizo and co‐authors kicked off the issue by discussing the important role that structural biology has played in uncovering the mechanisms underlying neurotransmitter release while highlighting the limitations of this approach [13]. In the second review article in the issue, Frédéric Pincet and co‐authors used experimental data and simple physics and chemistry models to analyse the kinetics and energetics of the entire fusion process [14]. Concluding the issue, Shen Wang and Cong Ma described the current understanding of how the Munc18‐1 and Munc13‐1 proteins guide neuronal SNARE complex assembly [15].

We would like to express our sincere gratitude to the guest editors and authors of all of the In the Limelight issues published in the journal, and encourage everyone to read this excellent content.

### Research Protocols

In 2022, we introduced a new article type: Research Protocols. These articles describe an experimental protocol in more detail than that typically included in the methods section of a research article, providing a comprehensive list of all required reagents, a step‐by‐step procedure and a troubleshooting section. We hope that the publication of Research Protocols will play a part in countering the life sciences reproducibility crisis, by giving researchers a platform to share their experiences and expertise in how to reliably perform technically challenging protocols. All Research Protocols undergo vigorous peer review to ensure that they make a meaningful contribution to the scientific community and are based on robust methodology, sound ethical standards and careful interpretation of data. We are pleased to announce that we have published our first Research Protocols on a method for validating and mapping protein–protein interactions and Expansion microscopy‐based imaging for visualisation of mitochondria in *Drosophila* ovarian germline stem cells, respectively [16, 17]. We encourage all researchers with an experimental procedure that they would like to share with the community to submit a Research Protocol to *FEBS Open Bio*.

### Journal prizes

The 2022 *FEBS Open Bio* Article Prize was awarded to Sofia Lovestam, the first author of the outstanding paper “Seeded assembly *in vitro* does not replicate the structures of α‐synuclein filaments from multiple system atrophy,” published in *FEBS Open Bio* in 2021 [18]. The winning paper was selected by a jury comprised of three members of the journal's editorial board: Ivana Novak, Sandro Sonnino and Alex Wlodawer. We would like to extend our congratulations to Sofia, and encourage you all to read the winning article. Sofia and the winner of the 2021 Prize, Arpit Katiyar [19], were both invited to attend and present short talks at the 46th FEBS Congress held in Lisbon, Portugal in 2022.

*FEBS Open Bio* has been awarding a single poster prize at the annual FEBS Congress for several years, but in 2022, we started to award poster prizes at multiple international events. We are pleased to be able to acknowledge and reward excellent work by early career researchers, and extend our congratulations to everyone who won a *FEBS Open Bio* poster prize in 2022 and previous years.

### Editorial board meeting

In October 2022, we held the first in‐person *FEBS Open Bio* editorial board meeting in Seville, Spain. This meeting was a very long time in the making: we had originally planned to hold a physical meeting in 2020, but were forced to change it to a virtual meeting due to the COVID‐19 pandemic. Last year's in‐person meeting was very well attended, with over 20 members of our editorial board, the editorial office staff and representatives from Wiley and the FEBS Publication Committee. We engaged in lively discussions on journal performance, the perils of paper mill submissions and how to continue to meet the needs of the scientific community through new innovations, and we expect that some of the proposals discussed will bear fruit in 2023.

We were delighted to meet so many friends and colleagues in person after two years of virtual meetings and e‐mail exchanges. We hope that everyone who attended had an enjoyable and enlightening time in Seville, and we thank the participants for their contributions to the discussions.

## Looking forward to 2023

The coming year promises to be just as exciting as 2022, with several new ‘In the Limelight’ issues on the way; these include issues focussing on Alzheimer's disease and glycosphingolipids in human diseases. In addition, we are planning to publish a special issue of articles authored by recipients of FEBS fellowships, to commemorate the FEBS fellows meeting that was held in Lisbon last year. We will also continue to publish profiles with our editors as part of our ‘an open chat with…’ series, with multiple interviews in the works.

We are also very much looking forward to this year's FEBS Congress, which will be held in Tours, France from 8th to 12th July 2023. For the first time ever, *FEBS Open Bio* and the other FEBS Press journals have contributed to the organisation of the event, and we are proud to announce that there are three sessions organised by *FEBS Open Bio* and chaired by our editors: (a) Protein life cycle I: localisation, dynamics, functioning, chaired by Irene Díaz‐Moreno; (b) Cell metabolism and stress, chaired by Laszlo Nagy; and (c) RNA biology, chaired by Cornelia De Moor. In addition, this year's Congress will feature the first ever *FEBS Open Bio* Lecture, which in 2023 will be given by our editor Jose Rizo‐Rey on the topic of molecular mechanisms underlying neurotransmitter release and its regulation. We strongly encourage everyone to consider registering for this year's Congress here: https://2023.febscongress.org/registration.

In closing, we would like to thank all of our editors, authors, colleagues, readers and reviewers for their invaluable help and support in 2022. We look forward to working with them in 2023.

## Conflict of interest

The authors declare no conflict of interest.

## Author contributions

DEW and MAR wrote the editorial.

## References

[1] Martinez‐Fabregas J , Tamargo‐Azpilicueta J , Diaz‐Moreno I . Lysosomes: multifunctional compartments ruled by a complex regulatory network. FEBS Open Bio. 2022;12:758–74. 10.1002/2211-5463.13387 PMC8972048 35218162

[2] Kos J , Mitrović A , Nanut MP , Pišlar A . Lysosomal peptidases—intriguing roles in cancer progression and neurodegeneration. FEBS Open Bio. 2022;12:708–38. 10.1002/2211-5463.13372 PMC8972049 35067006

[3] Carosi JM , Fourrier C , Bensalem J , Sargeant TJ . The mTOR–lysosome axis at the centre of ageing. FEBS Open Bio. 2021;12:739–57. 10.1002/2211-5463.13347 PMC8972043 34878722

[4] Reinheckel T , Tholen M . Low‐level lysosomal membrane permeabilization for limited release and sublethal functions of cathepsin proteases in the cytosol and nucleus. FEBS Open Bio. 2022;12:694–707. 10.1002/2211-5463.13385 PMC8972055 35203107

[5] Watts C . Lysosomes and lysosome‐related organelles in immune responses. FEBS Open Bio. 2022;12:678–93. 10.1002/2211-5463.13388 PMC8972042 35220694

[6] Francisco‐Velilla R , Embarc‐Buh A , Abellan S , Martinez‐Salas E . Picornavirus translation strategies. FEBS Open Bio. 2022;12:1125–41. 10.1002/2211-5463.13400 PMC9157412 35313388

[7] Reyes A , Navarro AJ , Diethelm‐Varela B , Kalergis AM , González PA . Is there a role for HSF1 in viral infections? FEBS Open Bio. 2022;12:1112–24. 10.1002/2211-5463.13419 PMC9157408 35485710

[8] Caceres CJ , Seibert B , Faccin FC , Cardenas‐Garcia S , Rajao DS , Perez DR . Influenza antivirals and animal models. FEBS Open Bio. 2022;12:1142–65. 10.1002/2211-5463.13416 PMC9157400 35451200

[9] Deymier S , Louvat C , Fiorini F , Cimarelli A . ISG20: an enigmatic antiviral RNase targeting multiple viruses. FEBS Open Bio. 2022;12:1096–111. 10.1002/2211-5463.13382 PMC9157404 35174977

[10] Frazier MN , Riccio AA , Wilson IM , Copeland WC , Stanley RE . Recent insights into the structure and function of coronavirus ribonucleases. FEBS Open Bio. 2022;12:1567–83. 10.1002/2211-5463.13414 PMC9110870 35445579

[11] Eriani G , Martin F . Viral and cellular translation during SARS‐CoV‐2 infection. FEBS Open Bio. 2022;12:1584–601. 10.1002/2211-5463.13413 PMC9110871 35429230

[12] Qiao S , Zhang S , Ge J , Wang X . Thee spike glycoprotein of highly pathogenic human coronaviruses: structural insights for understanding infection, evolution and inhibition. FEBS Open Bio. 2022;12:1602–22. 10.1002/2211-5463.13454 PMC9433818 35689514

[13] Rizo J , David G , Fealey ME , Jaczynska K . On the difficulties of characterizing weak protein interactions that are critical for neurotransmitter release. FEBS Open Bio. 2022;12:1912–38. 10.1002/2211-5463.13473 PMC9623538 35986639

[14] Wang S , Ma C . Neuronal SNARE complex assembly guided by Munc18‐1 and Munc13‐1. FEBS Open Bio. 2022;12:1939–57. 10.1002/2211-5463.13394 PMC9623535 35278279

[15] Mion D , Bunel L , Heo P , Pincet F . The beginning and the end of SNARE‐induced membrane fusion. FEBS Open Bio. 2022;12:1958–79. 10.1002/2211-5463.13447 PMC9623537 35622519

[16] Réthi‐Nagy Z , Ábrahám E , Lipinszki Z . GST‐IVTT pull‐down: a fast and versatile in vitro method for validating and mapping protein–protein interactions. FEBS Open Bio. 2022;12:1988–95. 10.1002/2211-5463.13485 PMC9623517 36102272

[17] Lin C‐H , Lin T‐Y , Hsu S‐C , Hsu H‐J . Expansion microscopy‐based imaging for visualization of mitochondria in Drosophila ovarian germline stem cells. FEBS Open Bio. 2022. 10.1002/2211-5463.13506 PMC9714352 36331359

[18] Lövestam S , Schweighauser M , Matsubara T , Murayama S , Tomita T , Ando T , et al. Seeded assembly in vitro does not replicate the structures of α‐synuclein filaments from multiple system atrophy. FEBS Open Bio. 2021;11:999–1013. 10.1002/2211-5463.13110 PMC8016116 33548114

[19] Katiyar A , Fujimoto M , Tan K , Kurashima A , Srivastava P , Okada M , et al. HSF1 is required for induction of mitochondrial chaperones during the mitochondrial unfolded protein response. FEBS Open Bio. 10.1002/2211-5463.12863 PMC7262932 32302062

